# Intramural Coronary Artery Course in Jatene Operation for Transposition of Great Arteries: Still a Challenge

**DOI:** 10.5935/1678-9741.20160017

**Published:** 2016

**Authors:** Marcelo Biscegli Jatene, Leonardo Augusto Miana

**Affiliations:** 1 Instituto do Coração do Hospital das Clínicas da Faculdade de Medicina da Universidade de São Paulo (InCor HC-FMUSP), São Paulo, SP, Brazil. E-mail: marcelo.jatene@incor.usp.br

The surgical treatment of transposition of the great arteries (TGA) is a well-established and routine procedure nowadays, the Jatene operation (arterial switch operation - ASO) being the treatment of choice for almost all cases of simple TGA and all other presentations, including TGA with ventricular septal defects, pulmonary stenosis and aortic arch anomalies.

In developing countries, issues like late presentation and lack of neonatal care infrastructure are problems faced on a daily basis. Certainly, that interferes with surgical results. It is also known that anatomical aspects impose morbidity for a child with TGA that requires an operation. Coronary artery anomalies are on the top of the list of anatomical abnormalities and can affect early and late results^[1]^.

Considering all of the coronary artery anomalies described, one of the most frightening types for surgical translocation are the coronaries with intramural course^[2]^. In this edition of the Brazilian Journal of Cardiovascular Surgery, Mishra et al.^[3]^ report an alternative technique to deal with intramural coronaries in TGA patients.

For a surgical team that is planning a Jatene operation, previous and detailed information about coronary arteries anatomy is essential for an adequate operative plan. Usually, transthoracic echocardiogram provides that information, but in some cases, the surgeon identifies the anomaly while operating, during the initial dissection of the great vessels or, more frequently, after opening and examining the aorta and the ostia.

Identifying a coronary intramural course during the operation is not a desired situation. The surgeon has to decide the best correction and strategy under such a great and unexpected pressure. In their paper, Mishra et al had to deal with this situation in every case, which affords more importance and relevance to the surgical team, considering the adequate results achieved^[3]^.

There are many surgical techniques described in the literature to treat intramural coronary arteries during a Jatene operation^[4]^. Almost all of them follow the philosophy of using the unroofing procedure for the intramural course and, often, separation of the coronary ostia, particularly when they arise very close to each other^[4]^. Then, right and left coronaries (after being unroofed) are translocated to different spots at the neoaorta.

The absence of preoperative diagnosis is not uncommon and may cause eventual lesion during coronary dissection in intramural cases^[4]^, mainly when the left coronary is committed by the intramural course. When the surgeon begins the ostial excision with no information or suspicious of an intramural course, the coronary can be damaged, with unpredictable surgical maneuvers to fix it. The use of an instrument to explore the coronary route is necessary when there is a possibility of coronary anomaly. The external view and inspection of a possible intramural course must be completed with a meticulous internal inspection and adequate definition of the trajectory and length of the intramural course. It is also safer and better to separate the coronaries when they are very close to each other and then reallocate them separately in a more favorable anatomical position in the neoaorta, avoiding kinking, stretching and torsion.

The technique described by Mishra et al.^[3]^, using intracoronary shunts inside the intramural course of the coronary artery, seems to be very useful, providing more safety in the unroofing procedure. We agree with the authors that we should have intracoronary shunts of different sizes available during a Jatene operation to provide an additional strategy to deal with such a challenging situation.

We shall emphasize that the Jatene operation in the presence of intramural coronary arteries is a high-risk operation^[4]^ and must be considered by a very well trained surgeon and surgical team, in a hospital with adequate experience and structure, in order to achieve the best result expected.
